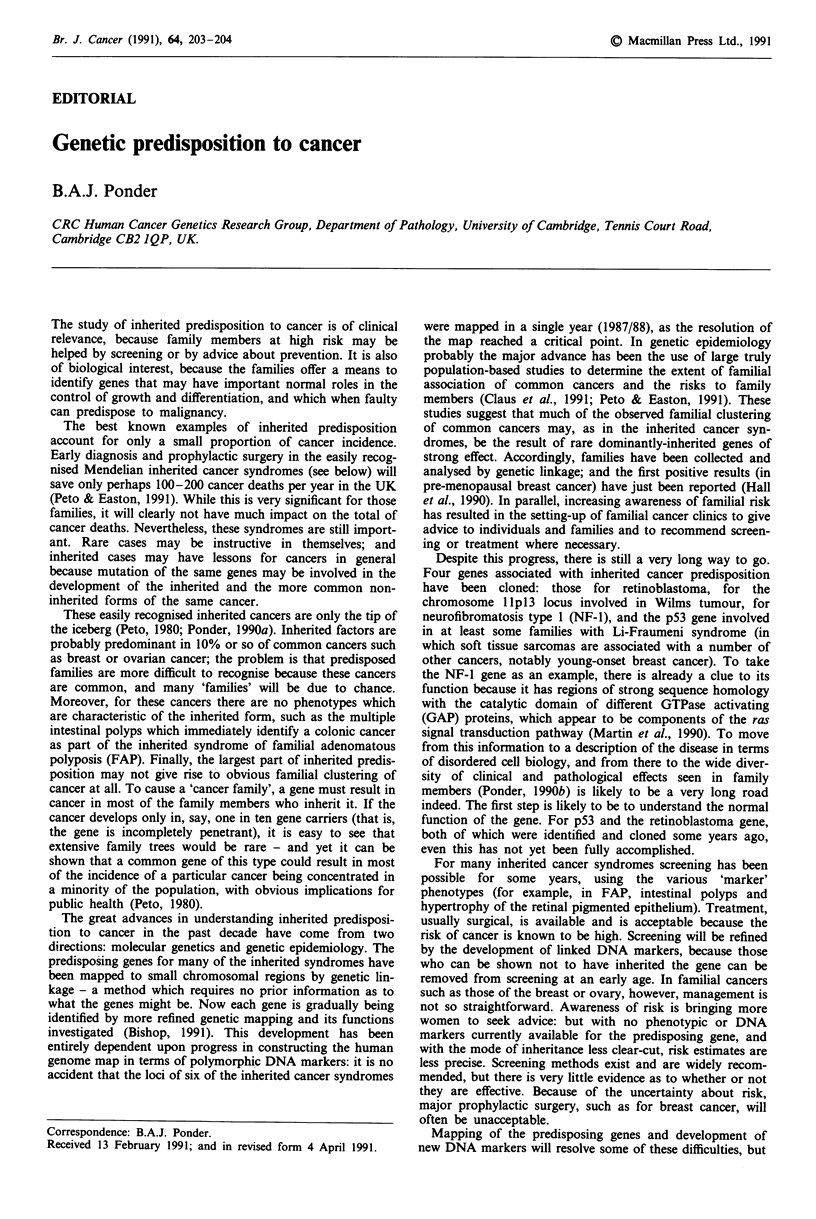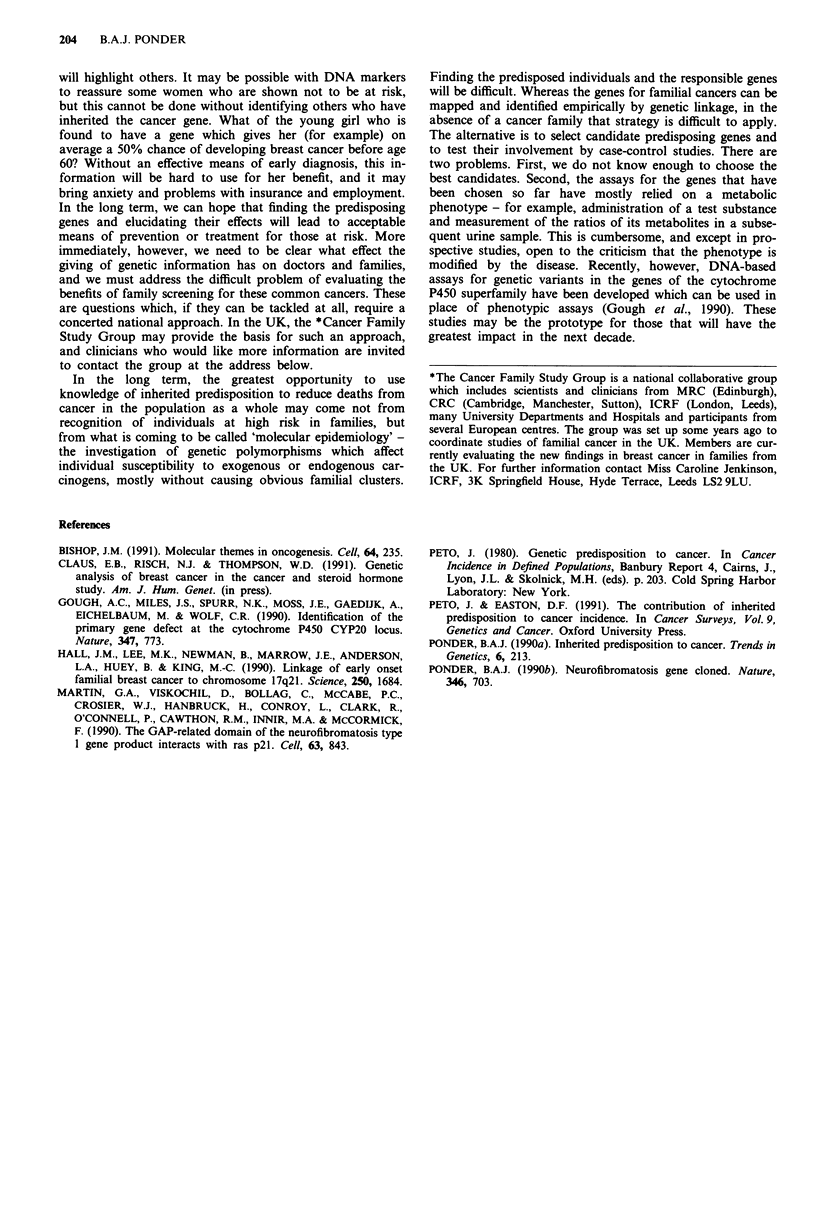# Genetic predisposition to cancer.

**DOI:** 10.1038/bjc.1991.275

**Published:** 1991-08

**Authors:** B. A. Ponder


 © Macmillan Press Ltd., 1991

EDITORIAL

### Genetic predisposition to cancer

B.A.J. Ponder

*CRC Human Cancer Genetics Research Group, Department of Pathology, University of Cambridge, Tennis Court Road, Cambridge CB2 1QP, UK.*

The study of inherited predisposition to cancer is of clinical relevance, because family members at high risk may be helped by screening or by advice about prevention. It is also of biological interest, because the families offer a means to identify genes that may have important normal roles in the control of growth and differentiation, and which when faulty can predispose to malignancy.

The best known examples of inherited predisposition account for only a small proportion of cancer incidence. Early diagnosis and prophylactic surgery in the easily recognised Mendelian inherited cancer syndromes (see below) will save only perhaps 100-200 cancer deaths per year in the UK (Peto & Easton, 1991). While this is very significant for those families, it will clearly not have much impact on the total of cancer deaths. Nevertheless, these syndromes are still important. Rare cases may be instructive in themselves; and inherited cases may have lessons for cancers in general because mutation of the same genes may be involved in the development of the inherited and the more common non-inherited forms of the same cancer.

These easily recognised inherited cancers are only the tip of the iceberg (Peto, 1980; Ponder, 1990*a*). Inherited factors are probably predominant in 10% or so of common cancers such as breast or ovarian cancer; the problem is that predisposed families are more difficult to recognise because these cancers are common, and many 'families' will be due to chance. Moreover, for these cancers there are no phenotypes which are characteristic of the inherited form, such as the multiple intestinal polyps which immediately identify a colonic cancer as part of the inherited syndrome of familial adenomatous polyposis (FAP). Finally, the largest part of inherited predisposition may not give rise to obvious familial clustering of cancer at all. To cause a 'cancer family', a gene must result in cancer in most of the family members who inherit it. If the cancer develops only in, say, one in ten gene carriers (that is, the gene is incompletely penetrant), it is easy to see that extensive family trees would be rare - and yet it can be shown that a common gene of this type could result in most of the incidence of a particular cancer being concentrated in a minority of the population, with obvious implications for public health (Peto, 1980).

The great advances in understanding inherited predisposition to cancer in the past decade have come from two directions: molecular genetics and genetic epidemiology. The predisposing genes for many of the inherited syndromes have been mapped to small chromosomal regions by genetic linkage - a method which requires no prior information as to what the genes might be. Now each gene is gradually being identified by more refined genetic mapping and its functions investigated (Bishop, 1991). This development has been entirely dependent upon progress in constructing the human genome map in terms of polymorphic DNA markers: it is no accident that the loci of six of the inherited cancer syndromes were mapped in a single year (1987/88), as the resolution of the map reached a critical point. In genetic epidemiology probably the major advance has been the use of large truly population-based studies to determine the extent of familial association of common cancers and the risks to family members (Claus *et al.*, 1991; Peto & Easton, 1991). These studies suggest that much of the observed familial clustering of common cancers may, as in the inherited cancer syndromes, be the result of rare dominantly-inherited genes of strong effect. Accordingly, families have been collected and analysed by genetic linkage; and the first positive results (in pre-menopausal breast cancer) have just been reported (Hall *et al.*, 1990). In parallel, increasing awareness of familial risk has resulted in the setting-up of familial cancer clinics to give advice to individuals and families and to recommend screening or treatment where necessary.

Despite this progress, there is still a very long way to go. Four genes associated with inherited cancer predisposition have been cloned: those for retinoblastoma, for the chromosome 11p13 locus involved in Wilms tumour, for neurofibromatosis type 1 (NF-1), and the p53 gene involved in at least some families with Li-Fraumeni syndrome (in which soft tissue sarcomas are associated with a number of other cancers, notably young-onset breast cancer). To take the NF-1 gene as an example, there is already a clue to its function because it has regions of strong sequence homology with the catalytic domain of different GTPase activating (GAP) proteins, which appear to be components of the *ras* signal transduction pathway (Martin *et al.*, 1990). To move from this information to a description of the disease in terms of disordered cell biology, and from there to the wide diversity of clinical and pathological effects seen in family members (Ponder, 1990*b*) is likely to be a very long road indeed. The first step is likely to be to understand the normal function of the gene. For p53 and the retinoblastoma gene, both of which were identified and cloned some years ago, even this has not yet been fully accomplished.

For many inherited cancer syndromes screening has been possible for some years, using the various 'marker' phenotypes (for example, in FAP, intestinal polyps and hypertrophy of the retinal pigmented epithelium). Treatment, usually surgical, is available and is acceptable because the risk of cancer is known to be high. Screening will be refined by the development of linked DNA markers, because those who can be shown not to have inherited the gene can be removed from screening at an early age. In familial cancers such as those of the breast or ovary, however, management is not so straightforward. Awareness of risk is bringing more women to seek advice: but with no phenotypic or DNA markers currently available for the predisposing gene, and with the mode of inheritance less clear-cut, risk estimates are less precise. Screening methods exist and are widely recommended, but there is very little evidence as to whether or not they are effective. Because of the uncertainty about risk, major prophylactic surgery, such as for breast cancer, will often be unacceptable.

Mapping of the predisposing genes and development of new DNA markers will resolve some of these difficulties, but

Correspondence: B.A.J. Ponder.
Received 13 February 1991; and in revised form 4 April 1991.

will highlight others. It may be possible with DNA markers to reassure some women who are shown not to be at risk, but this cannot be done without identifying others who have inherited the cancer gene. What of the young girl who is found to have a gene which gives her (for example) on average a 50% chance of developing breast cancer before age 60? Without an effective means of early diagnosis, this information will be hard to use for her benefit, and it may bring anxiety and problems with insurance and employment. In the long term, we can hope that finding the predisposing genes and elucidating their effects will lead to acceptable means of prevention or treatment for those at risk. More immediately, however, we need to be clear what effect the giving of genetic information has on doctors and families, and we must address the difficult problem of evaluating the benefits of family screening for these common cancers. These are questions which, if they can be tackled at all, require a concerted national approach. In the UK, the *Cancer Family Study Group may provide the basis for such an approach, and clinicians who would like more information are invited to contact the group at the address below.

In the long term, the greatest opportunity to use knowledge of inherited predisposition to reduce deaths from cancer in the population as a whole may come not from recognition of individuals at high risk in families, but from what is coming to be called 'molecular epidemiology' – the investigation of genetic polymorphisms which affect individual susceptibility to exogenous or endogenous carcinogens, mostly without causing obvious familial clusters. Finding the predisposed individuals and the responsible genes will be difficult. Whereas the genes for familial cancers can be mapped and identified empirically by genetic linkage, in the absence of a cancer family that strategy is difficult to apply. The alternative is to select candidate predisposing genes and to test their involvement by case-control studies. There are two problems. First, we do not know enough to choose the best candidates. Second, the assays for the genes that have been chosen so far have mostly relied on a metabolic phenotype – for example, administration of a test substance and measurement of the ratios of its metabolites in a subsequent urine sample. This is cumbersome, and except in prospective studies, open to the criticism that the phenotype is modified by the disease. Recently, however, DNA-based assays for genetic variants in the genes of the cytochrome P450 superfamily have been developed which can be used in place of phenotypic assays (Gough *et al.*, 1990). These studies may be the prototype for those that will have the greatest impact in the next decade.

*The Cancer Family Study Group is a national collaborative group which includes scientists and clinicians from MRC (Edinburgh), CRC (Cambridge, Manchester, Sutton), ICRF (London, Leeds), many University Departments and Hospitals and participants from several European centres. The group was set up some years ago to coordinate studies of familial cancer in the UK. Members are currently evaluating the new findings in breast cancer in families from the UK. For further information contact Miss Caroline Jenkinson, ICRF, 3K Springfield House, Hyde Terrace, Leeds LS2 9LU.

### References

BISHOP, J.M. (1991). Molecular themes in oncogenesis. *Cell*, **64**, 235.

CLAUS, E.B., RISCH, N.J. & THOMPSON, W.D. (1991). Genetic analysis of breast cancer in the cancer and steroid hormone study. *Am. J. Hum. Genet.* (in press).

GOUGH, A.C., MILES, J.S., SPURR, N.K., MOSS, J.E., GAEDIJK, A., EICHELBAUM, M. & WOLF, C.R. (1990). Identification of the primary gene defect at the cytochrome P450 CYP20 locus. *Nature*, **347**, 773.

HALL, J.M., LEE, M.K., NEWMAN, B., MARROW, J.E., ANDERSON, L.A., HUEY, B. & KING, M.-C. (1990). Linkage of early onset familial breast cancer to chromosome 17q21. *Science*, **250**, 1684.

MARTIN, G.A., VISKOCHIL, D., BOLLAG, C., MCCABE, P.C., CROSIER, W.J., HANBRUCK, H., CONROY, L., CLARK, R., O'CONNELL, P., CAWTHON, R.M., INNIR, M.A. & MCCORMICK, F. (1990). The GAP-related domain of the neurofibromatosis type 1 gene product interacts with ras p21. *Cell*, **63**, 843.

PETO, J. (1980). Genetic predisposition to cancer. In *Cancer Incidence in Defined Populations*, Banbury Report 4, Cairns, J., Lyon, J.L. & Skolnick, M.H. (eds). p. 203. Cold Spring Harbor Laboratory: New York.

PETO, J. & EASTON, D.F. (1991). The contribution of inherited predisposition to cancer incidence. In *Cancer Surveys, Vol. 9, Genetics and Cancer*. Oxford University Press.

PONDER, B.A.J. (1990*a*). Inherited predisposition to cancer. *Trends in Genetics*, **6**, 213.

PONDER, B.A.J. (1990*b*). Neurofibromatosis gene cloned. *Nature*, **346**, 703.